# Spatial validation of acoustic individual identification models without ground truths: a case study with the cao-vit gibbon population

**DOI:** 10.7717/peerj.20655

**Published:** 2026-03-02

**Authors:** Paul Best, Angela Dassow, Arik Kershenbaum, Tho Duc Nguyen, Megan Pogson, Aishwarya Maheshwari, Ricard Marxer

**Affiliations:** 1 Laboratoire d’Informatique des Systèmes, Université de Toulon, Toulon, France; 2 Institue of Language Communication and the Brain, Marseille, France; 3 Biology Department, Carthage College, Kenosha, WI, United States of America; 4 Girton College and Department of Zoology, University of Cambridge, Cambridge, United Kingdom; 5 Vietnam Programme, Fauna & Flora, Hanoi, Vietnam; 6 Graduate University of Science and Technology, Vietnam Academy of Science and Technology, Hanoi, Vietnam

**Keywords:** Bioacoustics, Acoustic Identification of Individuals (AIID), Deep learning, Gibbons

## Abstract

Technological progress has made bioacoustics an important tool for research in the ecology and behaviour of sound producing animals. Using an array of synchronised autonomous recorders, we can localise vocalising animals, and for certain species, computational models can acoustically identify individuals (AIID). Knowing both the precise location and identity of vocalising animals enables a more detailed interpretation of long-term bioacoustic data, but assessing the reliability of AIID models is often difficult, especially for populations that evolve over time. Annotated ground truth labels in test sets are commonly used, but they are often limited in size, and there can be a mismatch with the application data (for instance in case of a change in recording system). Here, we formalise a methodology to evaluate AIID models based on localised predictions, thus bypassing the need for ground truth labels. We demonstrate it on a case study with the critically endangered cao-vit gibbons (*Nomascus nasutus*). Using deep-learning, we develop an AIID model for male cao-vit gibbons. Then, we estimate its performance without any ground truths, using a new framework that relies on assumptions of territoriality. Empirical tests with simulated data show that this approach to no-ground-truth AIID evaluation is fairly reliable (0.05 of root mean square error between estimated and real accuracy for models with less than 30% of error rate), and specific flaws of performance estimation are described according to specific types of AIID errors. With this article, we demonstrate how spatialised data might help in the evaluation of AIID models for territorial species, both theoretically, and in practice with the cao-vit gibbon population.

## Introduction

Passive acoustic monitoring is an efficient approach for tracking vocal species, especially those inhabiting challenging or inaccessible terrains. Automated methods leverage the potential of long-term recordings by enabling systematic and reproducible analyses. In recent years, deep learning has significantly improved the reliability of these computational approaches across many fields, also helping to better detect and classify bioacoustic signals (Stowell, 2022).

One potential application of deep-learning in bioacoustics is for the acoustic identification of individuals (AIID), which could facilitate fine-scale population monitoring, and support both conservation and behavioural research. Linhart et al. (2022) review species with a potential for AIID and describe common challenges in solving the task, a major one being the lack of ground truth data. To address this, Knight et al. (2024) propose the use of identity proxies such as territoriality. However to the best of our knowledge, no study has yet established a specific framework for AIID without using focal recordings or prior knowledge of individual territories.

The eastern black-crested gibbon (*Nomascus nasutus*), also known as cao-vit gibbon, is a critically endangered species (Rawson et al., 2020) with a population of less than one hundred individuals, confined in a forest at the border between Vietnam and China (Fan et al., 2010; Lok, Xue-feng & Wu-jing, 2008). Their precarious status necessitates intense protection efforts, and monitoring their spatio-temporal presence is a key element of conservation strategies.

Gibbons (Hylobatidae) live in small groups and regularly produce stereotyped solo and duet songs thought to be a means of advertising pairbond and territorial defense (Cowlishaw, 1992). In many gibbon species, songs have been shown to carry individual information (enabling the identification of the emitter). Individual specific features were found either in female songs (Clink, Crofoot & Marshall, 2019; Haimoff & Gittins, 1985; Oyakawa, Koda & Sugiura, 2007; Dallmann & Geissmann, 2001; Terleph, Malaivijitnond & Reichard, 2015), or in male songs (Sun et al., 2011; Wanelik, Azis & Cheyne, 2013; Fan et al., 2011) including cao-vit gibbons’ (Feng et al., 2014; Wearn et al., 2024). Cao-vit gibbon songs consist of a succession of notes of four types sung by males: ‘aa’ notes, ‘boom’ notes, and frequency modulated notes(or ‘cao’), which are sometimes shortly followed by one or several ‘vit’ notes (Han et al., 2025). In the case of duet songs, these are answered by the females’ great call (Huaiqing, Huamei & Jiang, 2016). Cao-vit songs are often produced in sequences that can last half an hour or more, with the intensity (both in the amplitude of the sound and of the frequency modulation) progressively increasing until a stable point. The progression phase is referred to as the build-up (Wearn et al., 2024). In previous AIID studies dedicated to this species, the ‘cao’ notes were used to identify males (Feng et al., 2014; Wearn et al., 2024). The social structure of the cao-vit gibbons usually includes one adult male, two females, and other sub-adults, juveniles, and infants, which range in size from five to nine individuals (Wearn et al., 2024). In the large majority of cases, there is only one male that sings in a group, therefore we may use the name of the group to identify a ‘cao’ vocalisations.

With long-term acoustic recordings from devices spread-out over a wide area, vocal signatures represent an important opportunity to monitor species at a finer scale, for instance drawing territory boundaries and their evolution through time. However, it is impractical to analyse such long-term acoustic data manually, which motivates the development of automatic procedures. Machine learning is often chosen to solve such problems (Knight et al., 2024), either with supervised algorithms that are trained using annotated data, or with unsupervised algorithms that infer data categories without any labels. For instance, if all individuals in a population are known and samples of their vocalisations are available, one can employ supervised training to obtain an AIID model. On the other hand, if individual vocal signatures are not yet known, given an ensemble of vocalisations, an unsupervised model can identify clusters of similarity which might correspond to individual signatures. Several studies on gibbons have shown the potential of either unsupervised clustering algorithms to count singing individuals (Clink & Klinck, 2021; Wearn et al., 2024), or of supervised algorithms to detect vocalisations (Dufourq et al., 2021; Ruan et al., 2022) or classify song types (Zhou et al., 2023). A problem arises however with the evaluation of these models. Works with unsupervised learning or few-shot learning (Morfi et al., 2021) contribute to implementing AIID models with fewer or no ground truths, but practitioners still need an estimate of how reliable predictions are. Evaluating AIID models is currently principally based on annotated test sets, which are often limited in size, and are not necessarily representative of performance when recording systems vary.

In this paper, using estimated localisations of vocalising individuals, we demonstrate how the quality of identity predictions can be evaluated against different assumptions of territoriality, thus overcoming the common challenge of collecting the otherwise needed ground truths (Knight et al., 2024).

## Material & Methods

Gibbons were recorded in the wild from 17 July to 9 December 2022 in the Cao Vit Gibbon Species and Habitat Conservation Area (SHCA) in Trung Khanh District, Cao Bang Province, Vietnam. We used Wildlife Acoustics SM4 autonomous recording units (ARU) at 20 different stations. This recording protocal was conducted in strict compliance with and approved by the Institutional Animal Care and Use Committee (IACUC) at Carthage College (IACUC approval number 2022-01). Furthermore, there is no risk of impacting the privacy of people *via* incidental recording since recorders were placed in a nature reserve with restricted access.

**Figure 1:**
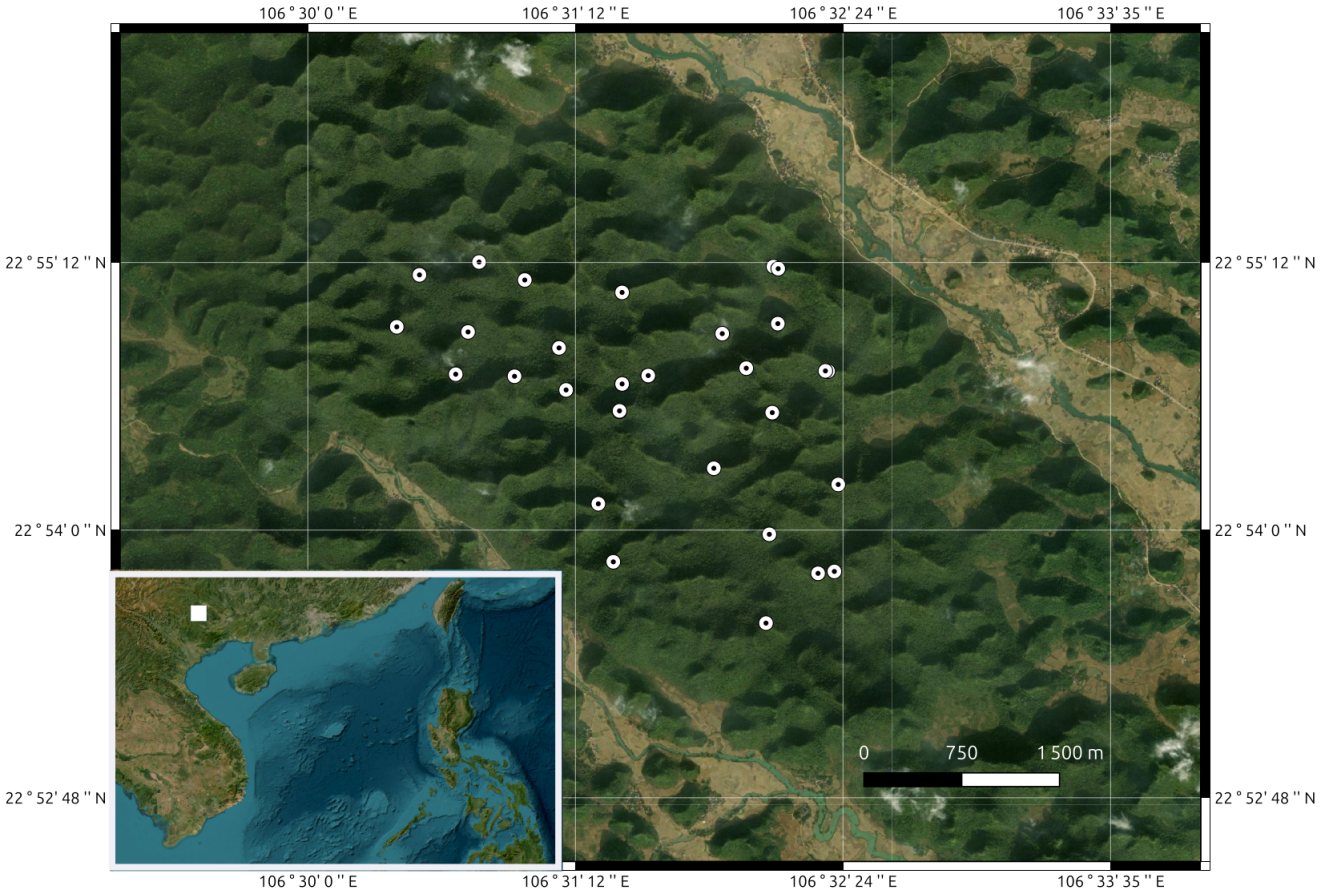
Satellite view of the study area in northern Vietnam, with markers denoting acoustic recorder stations. The map was made using the QGIS software with ESRI satellite data.

Station locations were at a mean distance of 1600 m from one another, with a range of 67-3400 m. All audio was recorded continuously through the day at a 16-bit, 16 kHz sampling rate. ARUs were time-synchronized using GPS time-synced receivers which synchronize the clocks across recording devices with an accuracy of within one millisecond. Recording units were placed at the peaks of mountain tops and ridges (Fig. 1) to maximise the potential for capturing cao-vit gibbon songs across previously identified family units (labelled AB, Q, TCN, AC, AA, and G2). Manually browsing recordings on Raven Pro 1.6 software (K. Lisa Yang Center for Conservation Bioacoustics at the Cornell Lab of Ornithology, 2023), 2,629 gibbon calls were then annotated to mark the times of male cao-vit vocalisations for use in training the detection algorithm.

**Figure 2:**
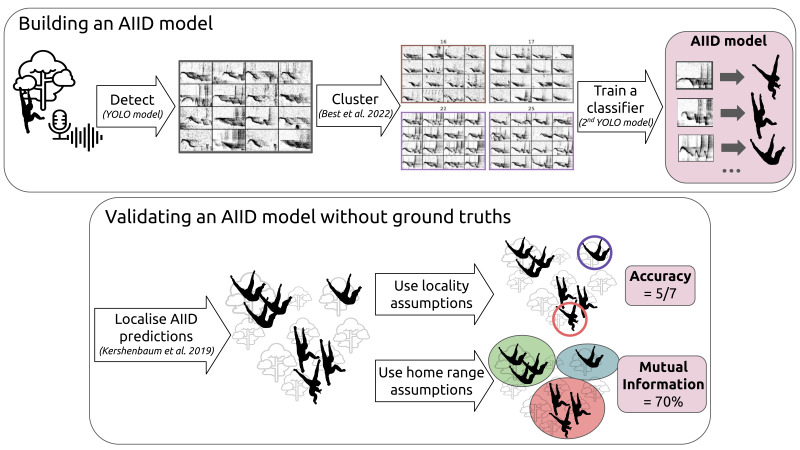
Study outline. We first demonstrate how to efficiently implement a model to acoustically identify individuals (top block), and then propose methodologies to validate this model based on different territoriality assumptions (bottom block). This framework is relatively generic, but here is applied to the identification of male cao-vit gibbons. The sequence of processing steps is shown by arrows, each having its dedicated section in the following Methods. For the locality assumptions procedure (top-right of the bottom panel), the red circle denotes a non-consistent ID, and the purple circle a non-specific ID.

For this study, we employed a range of computational tools to develop and test a model capable of identifying male gibbons from their ‘cao’ vocalisations. Figure 2 describes how the different steps come together, each having its associated part in this Methods section. The first parts of the methods (cao detection, feature extraction and clustering, and cao classification) apply existing methods to build an AIID model. We then propose two novel approaches to evaluate this model’s performance without ground truths. Both are based on localising vocalisation emitters (*i.e.* multilateration), but they rely on different assumptions: one assumes the locality of singers, while the other is based on known home ranges.

### Cao detection

We trained a You Only Look Once (YOLO) neural network (Redmon et al., 2016) to detect ‘cao’ vocalisations within spectrograms. Originally a standard in computer vision for object detection, the YOLO framework has also shown promising results in detecting non-human vocalisations in spectrograms (Coffey, Marx & Neumaier, 2019; Parcerisas et al., 2024; Escobar-Amado, Badiey & Wan, 2023).

The model was trained on 10 s spectrograms, iteratively optimised to predict bounding boxes around annotated ‘caos’ accurately. Spectrograms were generated from extracts including ‘cao’ annotations, using a 100 ms window size with no padding and 90% of overlap. A Mel-like bank of 64 filters, spanning frequencies from 1 kHz to 2 kHz (covering the fundamental frequency range of ‘cao’ vocalisations), was then applied. To reduce effects from stationary sounds (such as recorder’s self-noise and background noise), we subtracted the median of each frequency bin (Xie, Colonna & Zhang, 2021). Sampling negative examples is not necessary for this type of training since ‘caos’ are presented in large windows of 10 s, within which the model is penalised for detections besides the ‘cao’ bounding box, and other ‘caos’ should not be present since they are produced typically every 15 s.

Although the training set was relatively small (only 380 examples), the model reached satisfactory performance, achieving an average precision of 0.90 on the test set^1^ 1considering a detection as correct if its intersection over union (IoU) with an annotation is higher than 0.5(comprising 50 randomly sampled annotations). Apart from the spectrogram parameters, we used the YOLO v5 public implementation’s default settings for the model architecture, training and evaluation procedure (Jocher et al., 2022).

**Figure 3:**
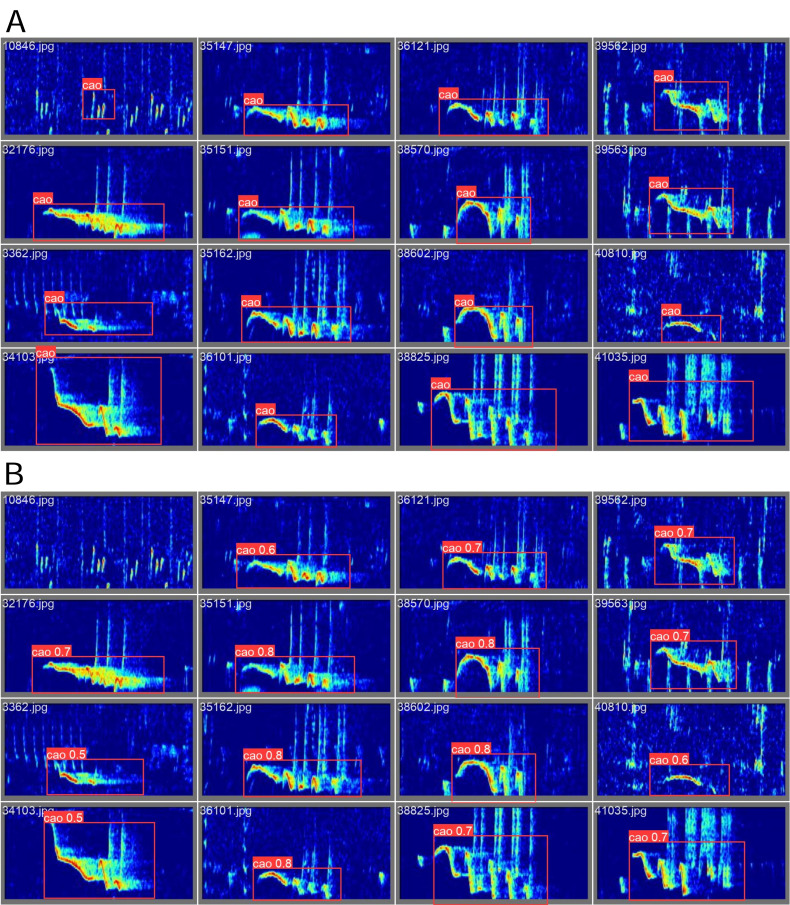
Examples of annotations (A) and predictions (B) from the test set after completion of the training.

Figure 3 shows examples of annotations and corresponding detections on the test set. The top left example shows a labelling error in which a ‘aa’ note was labelled as a cao. For this example the model did not detect anything, which is the desired behaviour. We used this detection model to extract ‘caos’ from long-term recordings, and later group them by similarity in order to reach an individual specific detection system.

Throughout all recordings, more than 41,000 ‘caos’ were detected, comprising 1,281 sequences of at least three ‘caos’ (considering ‘caos’ as part of the same sequence when less than 1 min apart). Among these, 520 sequences lasted eight minutes or longer, indicating that they had passed the build-up phase. For the clustering analysis, the more than 13,000 ‘caos’ that were not part of the song build-ups were retained.^2^ 2See the Supplementary Material text for motivations and implementation details of the sequence extraction and the song build-up filtering.

### Feature extraction and clustering

To cluster this set of vocalisations by acoustic similarity, we employed the procedure described in Best et al. (2023). This methodology proved its efficiency on a large variety of species for the categorisation of both vocal repertoires and individual signatures. It consists of generating embeddings with an auto-encoder neural network, trained to compress spectrograms into a lower-dimensional space while conserving enough information to reconstruct them (the auto-encoder can be a pre-trained one or one trained specifically). It is worth noting that to correctly reconstruct spectrograms, the embeddings should not be invariant to frequency or time shifts. Thus, despite being based on a convolutional architecture, the information of fundamental frequency (important for individual identification) is retained. Moreover, we used the frequency bin median normalisation to reduce the impact of site specific ambient noises.

For this study, we generated spectrograms of 2.5 s centred on ‘cao’ detections. This relatively short window helps us to ignore the ‘vit’ notes that sometimes occur after the ‘cao’ (since one individual can produce a varying number of these notes). The rest of settings for spectrograms computation are identical to those used in training the detection system. We thus trained an auto-encoder neural network to compress ‘cao’ spectrograms into a bottleneck of 256 dimensions, while maintaining enough information to reconstruct them (*i.e.* minimising the reconstruction loss). After training, the embeddings at the bottleneck were used as feature representation for describing vocalisations. Following Best et al. (2023), we clustered the data using a combination of manifold-based dimensionality reduction (UMAP algorithm; McInnes, Healy & Melville, 2018) and hierarchical density-based clustering (HDBSCAN algorithm; McInnes, Healy & Melville, 2018). Specifically, we employed UMAP to project embeddings into two dimensions, and HDBSCAN with a minimum cluster size of 100, and a requirement for at least three neighbouring points within a distance of 0.01 to define dense regions.

**Figure 4:**
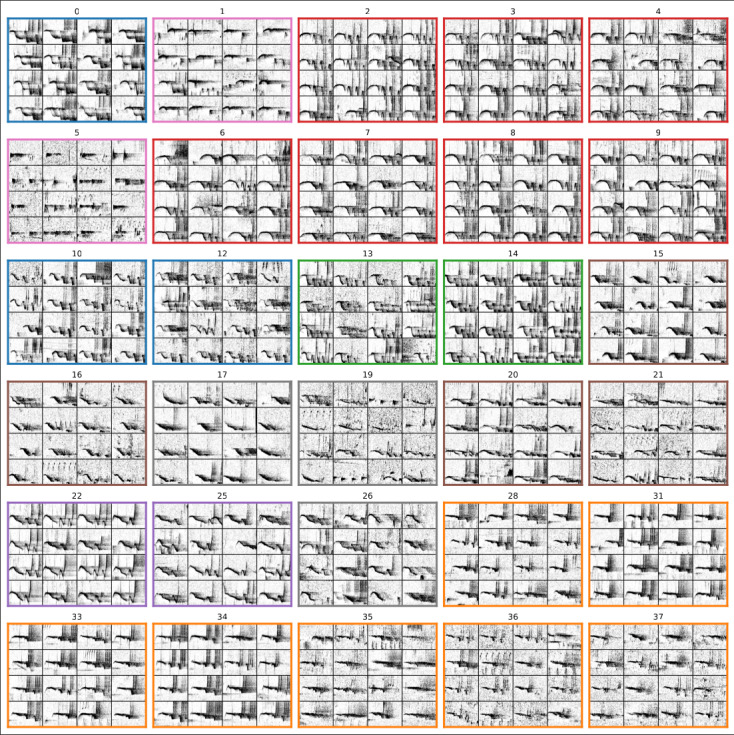
Spectrograms of ‘caos’ sampled from the different clusters (one box per cluster). Colours denote male attributions based on expert knowledge on their stereotypical frequency contours (AB in blue, TCN in orange, Q in green, AC in red, AA in purple, G2 in brown, false detections in pink, and mixed in grey).

Out of the 13,000 ‘caos’, 5,000 did not follow the set critera and were not clustered by HDBSCAN. The remaining vocalisations were grouped into 46 clusters of varying sizes (the largest being close to 500 ‘caos’). As a preliminary filter of noisy clusters, we tested if each cluster occurred in stations more than 2 km apart with a time delay of less than 30 min (gibbons travel speed were previously estimated between 400 and 1,000 m per hour Dunbar et al., 2019). Indeed, such occurrence indicates that vocalisations of different males are present in a cluster, and thus that the latter is not suited to be attributed a single male identity. Using a simple heuristic, this procedure allowed us to drop eight clusters, saving time in the necessary manual exploration / validation that follows. The 30 remaining clusters are shown in Fig. 4.

### Cao classification

For this study, clustering vocalisations primarily helped to gain efficiency in data annotation. Thousands of detected vocalisations were clustered by similarity of spectro-temporal features, such as frequency contours (Fig. 4), allowing an expert to efficiently browse and label these clusters (clusters were browsed exhaustively from spectrogram snipets on a standard file manager system). Some clusters contain vocalisations from only one male, enabling us to assign the group name based on prior knowledge of their unique frequency contours.^3^ 3Group labels are used to identify male singers here since there is only one male that sings in a group.Potential variations in recieved energy levels were also taken into account when labelling clusters (*e.g.*, clusters 1 and 5). Other clusters, composed mainly of false detections, were discarded in bulk. Occasionally, clusters reveal a mix of varying frequency contour shapes from different males; we categorise these as ‘mixed’ and exclude them from streamlined annotation. The assignment of labels to each cluster are shown by the border colours in Fig. 4.

The amount of vocalisations assigned to each male *via* clustering and manual validation (Fig. S1) is sufficient to train a neural network model for AIID, with each class gathering a minimum level of diversity (containing vocalisations from at least 30 different days). A typical approach to do so would be to train a classifier that, given a ‘cao’ spectrogram as input, outputs a gibbon ID (*i.e.*, a classification framework that relies on prior detection). However, since YOLO models are able to perform class-specific detection, we can simply train a new YOLO model (we will refer to it as ‘male ID YOLO’), which directly performs male-specific detections in long-term recordings, thus making a prior detection step unnecessary. We do so using identical spectrogram settings as for the preliminary ‘cao’ detector. Moreover, to better analyse songs, we added a sample of 200 female great calls (GC) to the training data, but this time with a single class regardless of individual identities because this kind of data is not yet available. To assess model performance, we built a test set with 20 randomly selected ‘caos’ of each class, excepting for great calls for which we selected 50.

### Multilateration

In order to further evaluate the performance of the implemented AIID model, we wish to make use of knowledge on the species’ territorial behaviour. For this purpose, we need estimates for the localisation of vocalising males. Following the method described in Kershenbaum, Owens & Waller (2019), for vocalisations that were detected on at least three recorders, we manually estimated the time difference between sound arrival across the recorders (recorders are time-synchronised *via* GPS). These time differences were then used to estimate the localisation of the sound source using standard methods (Rhinehart et al., 2020).

This analysis was conducted on a sample of 937 vocalisations that were both temporally and spatially spread out. Out of these, 254 fell outside the convex hull formed by the recorders positions, indicating potentially unreliable positioning, and were therefore excluded from subsequent analysis. The remaining vocalisations were assigned to individual emitters using the previously trained AIID model, with the distribution of these attributions provided in the Fig. S2.

YOLO models detect instances of specific classes but do not conduct classification in the strict sense. Thus, in 64 cases, the vocalisation used for multilateration did not trigger a detection and was therefore not assigned a male ID. It is probable that at least some of these vocalisations were from the song build-up phase, as they are much less stereotyped than the rest of the song (Wearn et al., 2024), and the AIID model was not trained to detect them.

### Using locality assumptions to estimate AIID accuracy

When individual territories are known and mutually exclusive, generating ground truths from locations is relatively straightforward (there is an unambiguous mapping between location and identity). However, when territories are not yet known or ephemeral, assessing a model quality is more challenging. To address this scenario, we introduce a framework to estimate an AIID model’s performance based on localised singing behaviour even when territories are not known. Notably, cao-vit gibbons select singing locations within their exclusive territory boundaries (Ma et al., 2020; Fan et al., 2010). The proposed approach is useful for a different scope than some previous studies on gibbon AIID (Clink & Klinck, 2021; Wearn et al., 2024) which tested the performance of unsupervised algorithms against manually annotated labels. Here, we measure the performance of a supervised classifier, trained with known labels even if the annotation process was accelerated *via* unsupervised methods, against locality assumptions that do not involve any labelling.

Table 1 describes the different locality assumptions proposed, and how they are interpreted in order to discriminate correct and erroneous AIID predictions. Specifically, we propose two complementary metrics: specificity and consistency. Besides the formulation of Table 1, an illustration of the two types of errors they measure is shown in Fig. 2. Also, the procedure to count presumably correct predictions, which are finally normalised by the total number of vocalizations, is described in the Supplementary Material Algorithm 1.

**Table 1:** Description of locality assumptions used to validate an AIID model without ground truths. These assumptions only need to be valid for a given temporal window (here it was fixed to one day).

Metric	Assumption	Description	Expected behaviour of AIID model	Identification of correct AIID predictions
**Consistency**	Minimal inter-individual distance	Individuals will not sing close to one another	Calls close to each other are associated to the same individual	Calls that are of the same ID as the majority of calls within their close radius
**Specificity**	Maximal intra-individual distance	An individual will not sing in far apart locations	Calls far from each other are associated to different individuals	Calls that have more identical ID in their close radius than outside of it.

With this method, we estimate a model’s quality by relying solely on its own predictions, raising questions about the reliability of the approach. To address this, we simulated data and compared these metrics to the ‘true model accuracy’ usually computed when ground truths are available. Each simulation begins by generating theoretical ground truths, randomly sampling *N* points from *n* possible ID classes. We then corrupt these ground truths to emulate an AIID model prediction. We modelled two types of errors: systematic confusions between two classes (by randomly reassigning all predictions of an individual to a different class with a probability *q*), and isolated model mistakes (by replacing a prediction by another one drawn from $[ \frac{n}{2} , \frac{3n}{2} ]$ with a probability of *p*). These two types of errors are also interpretable in AIID terms: over- and under-estimating population sizes. Systematic confusion between two classes correspond to an underestimation of the population, and half of the isolated mistakes, when the new class drawn is higher than *n*, correspond to an overestimation.

To understand the general behaviour of the approach under varying experimental conditions, we exhaustively sampled values of *N* from 100 to 1,000, values of *n* from 5 to 20, values of *q* from 0.1 to 0.5, and values of *p* from 0.1 to 1 (yielding 3,000 runs). After testing the approach on simulated data, we used it to estimate the quality of our cao-vit gibbon AIID model, considering that during a given day, calls of a single individual should be located within a 100 m radius (this threshold value was chosen empirically from the inspection of several days, measuring intra- and inter-individual distances). Only calls during days with at least one other call within this radius were considered to evaluate the consistency, and only calls during days with at least one other call outside of this radius were considered to measure the specificity. We also ran this evaluation with random ID predictions to have a baseline of comparison.

### Using home ranges to validate AIIDs

Sometimes, during passive acoustics monitoring, the home ranges of the target species are already known. When these home ranges are mutually exclusive, they can be used to infer a target ID from a localised vocalisation, thus generating ground truths for measuring an AIID model’s accuracy. However, there are cases when home ranges overlap, cases for which we propose a novel approach to validate an AIID model.

**Figure 5:**
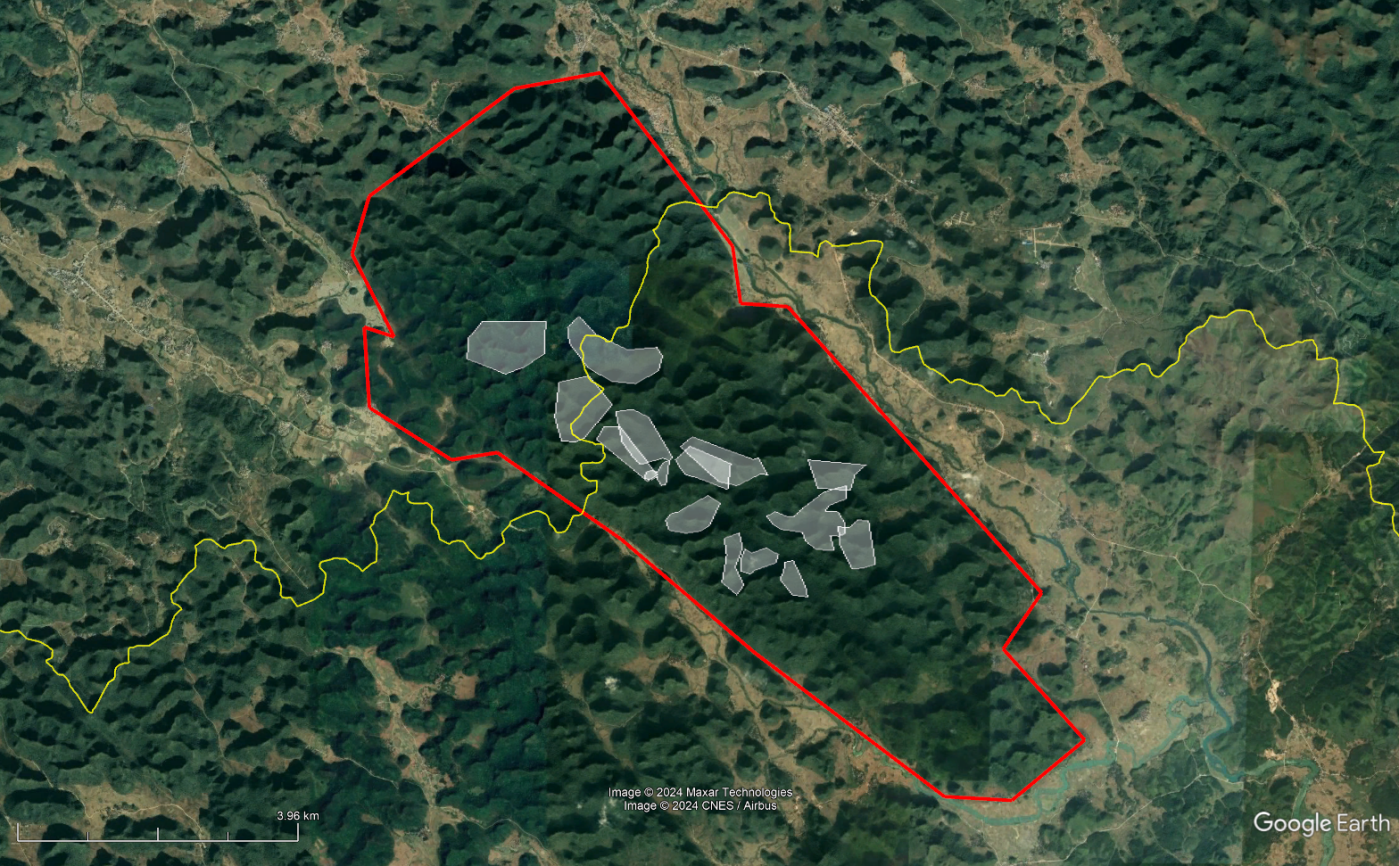
Approximation of the cao-vit gibbon home ranges from visual observations. This satellite view is copyrighted by Google Earth, Maxar Technologies 2024, and CNES / Airbus 2024.

The locally recruited Cao Vit Gibbon Conservation Team, responsible for monitoring and protecting the gibbon population, has collected observational data to estimate the home ranges of cao-vit gibbon family groups within the conservation area. Here, home range is defined as the territory in which a family group of gibbons carries out most of its activities (Bartlett, Light & Brockelman, 2016). During seasons immediately preceding acoustic data collection, the conservation team manually recorded visual and acoustic gibbon observations from each recording station for approximately two weeks each month, along with any opportunistic observations made between stations. These were then digitised using the Gaia software (Durham University) and integrated to generate convex polygons representing each approximate home range by Fauna & Flora International Vietnam Programme (Fig. 5). This method yielded 15 potential observed cao-vit gibbon group home ranges, of which three extend partly or fully across the Chinese border in the Bangliang Nature Reserve and have thus been excluded from the analysis.

We compared the locations of the multilaterated ‘caos’ from different groups to the observed home ranges by estimating the home ranges directly from the spatial distribution of vocalisations. We performed a kernel density estimation on the vocalisation locations for each group separately, using the MASS package in R (Ripley et al., 2013) with a bandwidth of 1,000 m and 50 grid points in each direction across the whole map. Only ‘caos’ with a group classification confidence >0.75 (from the YOLO network) were included. This gave for each group a 50 × 50 raster of the estimated probabilities that this group would be calling from each spatial location. Within each home range, kernel probabilities of raster pixels were summed for each gibbon group separately. This gave an estimate of the relative likelihood of each group calling in that home range, and the diversity of these relative likelihoods within that home range was calculated using the power law coefficient estimator for Shannon entropy (Kershenbaum et al., 2021) (*P*_*i*_ ∝ *i*^*k*^ with *k* the power law coefficient, and *P*_*i*_ the probability of group *i* ranked in descending order). A highly negative power law coefficient indicates that one group’s signature dominates the calls within a particular home range, whereas a coefficient close to zero indicates that the gibbon group probabilities in that home range are similar. This approach provides insights into the heterogeneity or homogeneity of AIID predictions within a given home range, serving as an indication of the model’s performance.

As a secondary method for measuring AIID performance, for each home range we identified the group with the highest estimated relative likelihood (summed kernel probability) for that home range, and assigned that group as the dominant (most likely) group in that home range. We then calculated the normalised mutual information between home range and dominant group identity, as formalised by Eq. (1) where *n*_*c*_ is the number of ‘caos’ attributed to group *c*, *n*_*k*_ is the number of ‘caos’ assigned to home range *k*, *n*_*k*,*c*_ is the number of ‘caos’ from group *c* in home range *k*, and *N* is the total number of ‘caos’. A normalised mutual information close to 1 indicates a strong association between each home range and a particular gibbon group, while a value close to 0 suggests no particular association between the two.

(1) \begin{eqnarray*}NMI= \frac{\sum _{k,c}{n}_{k,c}\log \nolimits \frac{N\cdot {n}_{k,c}}{{n}_{k}\cdot {n}_{c}} }{\sqrt{(\sum _{k}{n}_{k}\log \nolimits \frac{{n}_{k}}{N} )(\sum _{c}{n}_{c}\log \nolimits \frac{{n}_{c}}{N} )}} .\end{eqnarray*}

For both the power law coefficient and the normalised mutual information, we performed an exact test, carrying out 10,000 iterations in which the assignment of male ID to multilaterated point was randomised. This provides a baseline for comparing the AIID model’s performance, allowing us to compare the randomised null distributions of the two metrics with the observed metrics to calculate *p*-values.

## Results

### Clustering and classification

**Figure 6:**
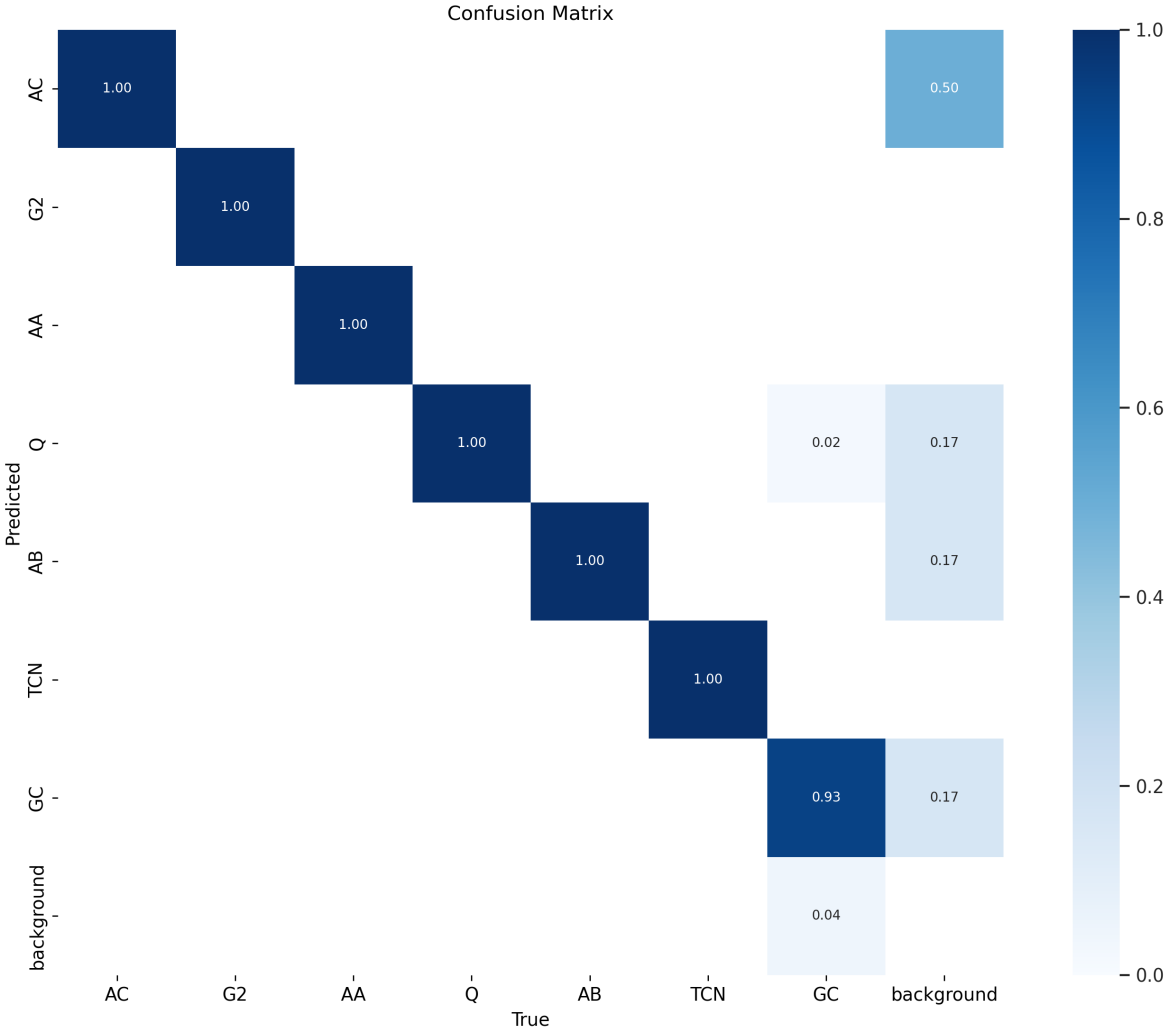
Confusion matrix of the male ID YOLO model on the test set.

The labelled vocalisations obtained *via* clustering and manual validation were used to train a male ID YOLO neural network, with the model’s performance evaluated on the test set presented as a confusion matrix (Fig. 6).

 Performance results shown in the confusion matrix indicate a largely positive outcome (mAP@0.5 = 0.98), with no misclassification between any of the classes, except for a single great-call incorrectly attributed to male Q. Nonetheless, these results do not guarantee correct male identifications, but only a correct match with the cluster attributions generated by the semi-automatic procedure. This motivated a supplementary validation described in the following sections.

### AIID accuracy estimation from locality assumptions

**Figure 7:**
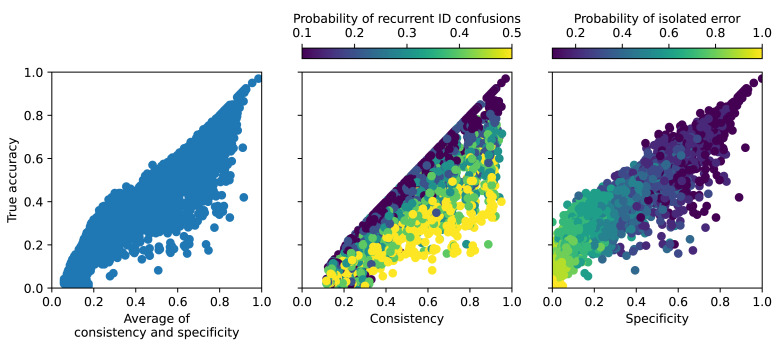
Real accuracy as a function of estimated metrics measured from simulated data, showing the correlation between the two. Each point corresponds to a simulated estimation of model quality, correct estimations being on the *y* = *x* line.

In Fig. 7, for the 3,000 simulated runs of AIID evaluation, we report the true accuracy as a function of estimated quality metrics, all derived from the same dataset. For this simulation, we can make abstraction of the spatialisation, considering points outside of the close radius as those with different theoretical ground truths, and points inside of the close radius as those with identical theoretical ground truths.

Figure 7 illustrates how specificity and consistency are each impacted differently by specific types of AIID errors. Consistency is more sensitive to systematic confusions between two individuals (middle panel of Fig. 7), which yield an overestimation of model accuracy. Indeed, if the majority of predictions are misclassified to the same ID, they will be mistakenly considered correct by the consistency metric (Table 1). As for the specificity, it is more sensitive to isolated errors (right panel of Fig. 7), which yield an underestimation of model accuracy. Indeed, with an increase of random errors, the probability of having more calls of the same ID outside of the close radius than inside of it increases, in which case a call would be falsely counted as a non-specific (Table 1).

Across the simulation settings, the average between consistency and specificity gives the most robust estimation of the real model accuracy, with a root mean square error (RMSE) of 0.105. Instead of averaging consistency and specificity, we also tried counting errors for each sample that was either not specific or not consistent, and using such count yielded a RMSE of 0.111.

The model accuracy estimation becomes increasingly challenging as accuracy decreases, which is anticipated since error detection relies on other predictions being correct. Notably, for simulations where the true model accuracy exceeds 0.7, the estimations are twice closer to true values (RMSE = 0.052 for the average of consistency and specificity, and RMSE = 0.044 for the count of both consistent and specific predictions).

Having assessed the reliability of estimating accuracy using consistency and specificity, we used this method to measure the performance of our AIID model (Table 2). To establish a baseline for comparison, we also ran this evaluation on random predictions for gibbon IDs, conducting this analysis over 100 trials. The resulting scores from these random predictions are reported in Table 2, offering a reference point to contextualise the model’s performance against chance-level outcomes.

**Table 2:** Performance metrics of the AIID model, estimated using the spatialisation of vocalisation joint with assumptions of territoriality. The baseline performance from random predictions is estimated over 100 runs and the standard deviation is given.

Metric	AIID Model	Random ID prediction
Specificity	0.70 (*N* = 415)	0.37 ± 0.08
Consistency	0.84 (*N* = 569)	0.58 ± 0.10
Accuracy	0.77	0.45 ± 0.06

### Agreement between AIIDs and home ranges

**Figure 8:**
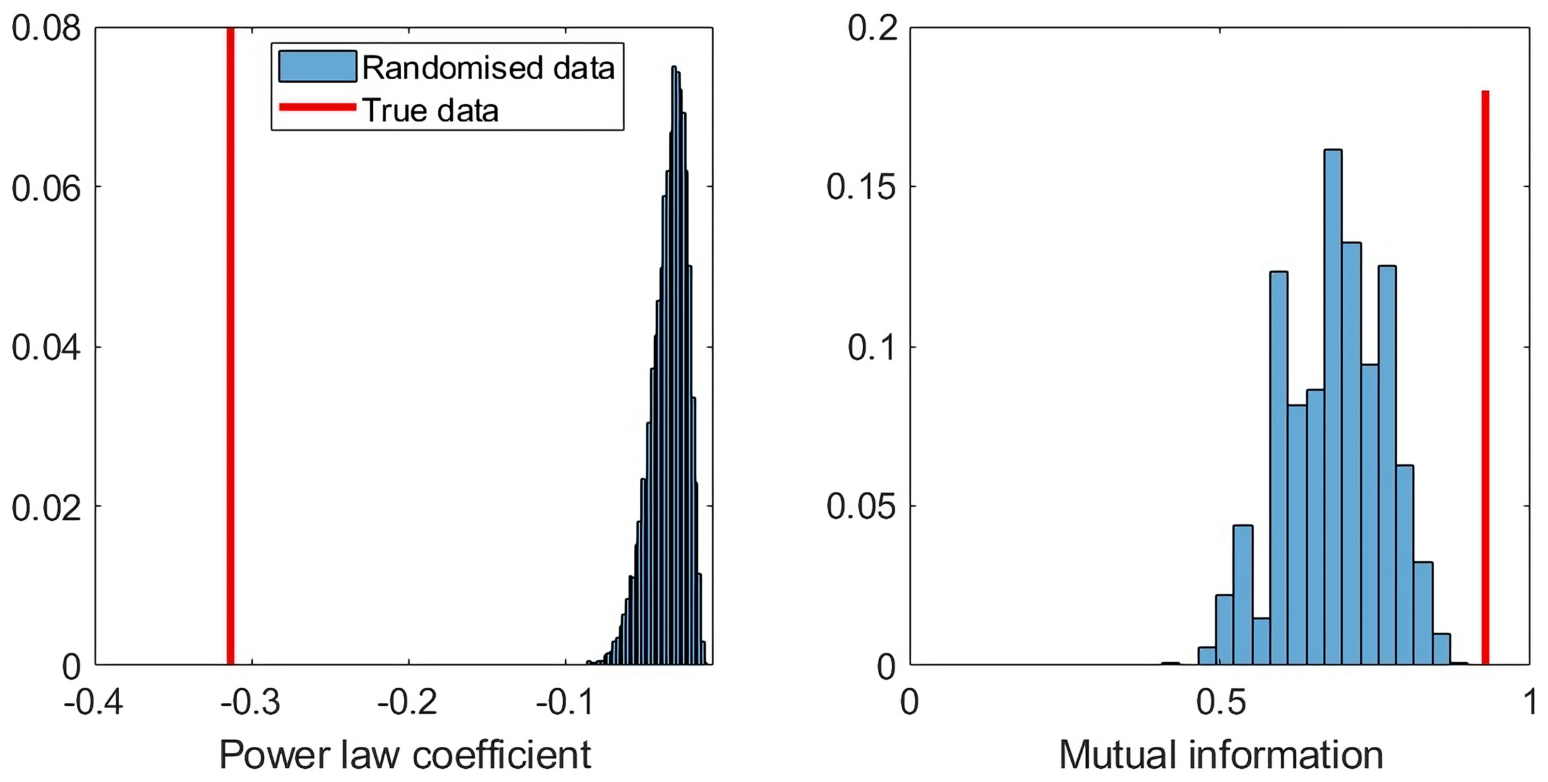
Exact test results for call type diversity (power law coefficient), and normalised mutual information with home range.

The diversity of call probabilities as given by the mean power law coefficient *k* =  − 0.313 within home ranges was significantly less than that expected in a randomised null population (mean *k* =  − 0.034, *p* < 0.001; Fig. 8). This finding indicates that home ranges were far more likely to contain calls from a specific male gibbon rather than a random distribution. The normalised mutual information between home range and most likely gibbon group was 0.93, which was significantly higher than that expected by chance (mean NMI = 0.69, *p* < 0.001).

## Discussion & Conclusion

In this study, we illustrate how computational bioacoustics might help in the conversation of a globally endangered species, with the case study of automatically identifying cao-vit gibbons’ vocalisations. Using only passive acoustic material, we outline a process for efficiently gathering annotation and training a classifier for individual acoustic identification.

In the past decades, AIID has gained interest from the bioacoustic community for its potential efficiency compared to visual or marking methods. In fact, numerous vertebrate species produce individual specific vocalisations, whether fish (Amorim & Vasconcelos, 2008), amphibians (Bee & Gerhardt, 2001), birds (Terry, Peake & McGregor, 2005), or mammals (Taylor & Reby, 2010). However, the labor-intensive task of labelling data, essential for both training and testing AIID systems, remains a significant challenge, as is common across deep-learning-based bioacoustic applications (Stowell, 2022). Especially for AIID, the fact that populations are not stable over long periods of time (individuals appear and disappear) implies that labelled data must be constantly updated to provide a true measure of a model’s reliability.

To address the lack of annotations, researchers often resort to unsupervised algorithms (Clink & Klinck, 2021; Wearn et al., 2024; Knight et al., 2024; Sadhukhan, Root-Gutteridge & Habib, 2021). In line with prior studies (Best et al., 2023), our use of deep auto-encoder embeddings facilitated the clustering of vocalisations based on spectro-temporal features without requiring pre-existing labels. By combining these algorithmically generated categories with expert knowledge, we reach a sufficient amount of data to train a neural network model at detecting individual-specific vocalisations in spectrograms, efficiently and with minimal time investment. The trained model reached a mAP of 0.98 in recognising male cao-vit gibbon vocalisations on the test set. Most apparent errors occur with false positives, which could be due to the annotation method, as each 10-second windows presented was annotated only for the central vocalisation through the clustering procedure. While vocalisations from a single male typically occurs at intervals exceeding 10 s, neighbouring males’ calls might still occasionally occur within the same 10-second window. The high performance score here only indicates the match between predictions and labelled data, which is not necessarily a guarantee of performance in a real-world scenario.

Important efforts are being made in the bioacoustic community to cope with the lack of annotations, resorting to unsupervised learning but also to few-shot learning for instance (Morfi et al., 2021). With few or no annotation, these techniques are very much helpful to implement automatic models such as for AIID. However, practitioners still need a measure of how reliable the resulting models are, and so not just on cross-species benchmarks but also for their specific use case. This motivates researching no-ground-truth model evaluation procedures.

Numerous AIID systems have been developed to monitor territorial species (Laiolo et al., 2007; Budka, Wojas & Osiejuk, 2015; Clink & Klinck, 2021), and territoriality has been proposed as a potentially useful a priori knowledge to address the challenge of collecting individual-specific ground truth data (Knight et al., 2024). Moreover, recent technological developments are helping in the democratisation of spatialised bioacoustics: inferring a relatively precise position for the emitter of a vocalisation (Rhinehart et al., 2020; Heath et al., 2024; Kershenbaum, Owens & Waller, 2019). In this study, we combined territoriality with emitters’ location, proposing two specific methods to assess the quality of an AIID system without requiring labelled data.

For the locality assumptions based method, data simulations revealed that consistency and specificity are each corrupted by different types of errors. Systematic ID confusions tend to produce an over-optimistic consistency, while isolated errors result in a more pessimistic evaluation of specificity. We therefore propose averaging the two metrics to achieve a more reliable estimate of accuracy across conditions. Specificity and consistency are also complementary in the fact that they are each impacted differently in cases where territoriality assumptions are unverified. On the one hand, if two individuals sing closer than the supposed minimal inter-individual distance, the consistency metric will be underestimated (unless the model predicts the same identity for the individuals in the zone). On the other hand, if an individual sings at places further apart than the supposed maximal intra-individual distance, the specificity metric will be underestimated (unless the model predicts different identities for the different singing locations of the individual). Hence, a wrong assumption of minimal intra-individual distance and maximal intra-individual distance will lead to performance underestimation. It is also worth noting that localisation errors will lead to similar phenomena as locality assumptions being unverified. We tested also if more sample points would yield better accuracy estimates, but found no correlation between the two (*R*^2^ = 0.002), meaning that 100 spatialised AIID predictions are enough to evaluate a model.

Overall, our data simulations across various settings such as the number of individuals, the number of collected vocalisations, or the level of errors of the AIID model, demonstrates that the estimate is relatively close to the actual accuracy value, with a RMSE of 0.05 across conditions if the error rate is lower than 30%. Using this method, we thus estimate an accuracy of 0.77 for our cao-vit gibbon male identifier (as compared to 0.45 for a random baseline). Taking the potential accuracy estimation error into account, we expect the real accuracy of this AIID model to lie between 0.72 and 0.82. It is worth noting that this estimation could be over-pessimistic, as some predictions were potentially run on ‘caos’ that belong to song build-ups (these ‘caos’ show a great variability making the AIID challenging (Wearn et al., 2024), and were not shown during training).

As another alternative approach to validate an AIID model, we proposed to assess the level of agreement between known home ranges and ID predictions, which is applicable even in cases when home ranges overlap. Comparing observed gibbon home ranges to the model predictions of gibbon ID, we have shown that a small number of gibbon IDs are far more common in a particular home range than would be expected by chance. Predictions for more than one individual in a home range can be due to AIID errors, but also to territoty shifts and/or invasions. Also, for overlapping home ranges as is the case here, it is expected that more than one male is detected in them. Moreover, we found that dominant gibbon IDs share 96% of the information with the home range, also far higher than expected by chance. Again, such methodologies represent a significant opportunity to validate AIID models without ground truths, given that the individual home ranges of the population are known, and that data spatialisation is available.

Methodologies employed in this study, both for vocalisation clustering and AIID model validation, tackle the lack of annotation that computational bioacoustic research often suffers from. The illustrative application presented here, identifying male cao-vit gibbons from their vocal signatures, is successful. Furthermore, it is applicable to other species and research projects, as long as several assumptions are followed. First, vocal signatures exist and can be distinguishable (*e.g.*, from spectro-temporal features). The expert knowledge used here to attribute proposed categories to individuals is needed to conduct a supervised training as we did, but the spatial evaluation methodology we describe is very well applicable to unsupervised AIID model predictions. Second, the data is spatialised, and so at a sufficiently high resolution relative to inter-individual distances, and covers the territory of several individuals simultaneously. Third, individuals show some territoriality, either as being the only singer in a given radius and temporal window, or individual home ranges are known (whether they overlap or not).

Even in cases where all of these conditions are met, an important limitation of using territoriality to validate AIID models is the influence of background noise on classification systems. Indeed, background noise is sometimes used by machine learning systems as a proxy to solve bioacoustic classification tasks, rather than basing decisions on actual vocalisation features (Cauzinille et al., 2024). This is especially true when soundscapes are class specific, such as in classifying species that live in different habitats, or identifying individuals whose territories are exposed to specific acoustic sources. Hence, since the approach proposed here relies on territories to validate AIID models, it is presently unable to isolate such confounds. However, specific methods to cope with them have been proposed to alleviate this drawback (Stowell et al., 2019). Moreover, regarding the specific use-case of identifying male cao-vit gibbons presented here, the facts that we use a YOLO architecture (specifically designed to detect objects on images regardless of their surroundings), and that males’ home ranges overlap hence different males are to be detected in similar acoustic environments, suggest that background noise has a relatively low impact on our AIID model’s performance.

We anticipate that the methodologies outlined in this study, with their reasonable simplicity, efficiency and interpretability, will serve as valuable tools for passive bioacoustic research. Attributing individual identities to localised vocalisations will help increase our knowledge on the ecology and behaviour of wild-ranging animals, while minimising interference with them and demanding low human efforts.

## Supplemental Information

10.7717/peerj.20655/supp-1 Supplemental Information 1 Triangulated vocalisations with associated ID predictions Each line corresponds to a multilaterated vocalisation. Then, columns give data about the precise localisation estimation, booleans on whether it is in the convex hull or not, and the predicted group ID (e.g. TCN, Q, G2 ...).

10.7717/peerj.20655/supp-2 Supplemental Information 2 Checkpoint for the AIID model This compressed file contains weights for the trained YOLO model. These can be used with the YOLO v5 public implementation (https://github.com/ultralytics/yolov5) to run on new audio data.

10.7717/peerj.20655/supp-3 Supplemental Information 3 Kernel density estimator This R code runs a kernel density estimation for a given table of localised ID predictions, estimating the distribution of individuals in a given area, which can then allow to measure its agreement with known home ranges.

10.7717/peerj.20655/supp-4 Supplemental Information 4 Accuracy estimation simulation This code tests how well the proposed accuracy estimation based on locality assumptions performs. It iteratively creates AIID ground truths, corrupts them to fake error prone predictions, uses the predictions to estimate consistency and specificity based on locality assumptions, and then compares the estimated performance with the true accuracy.

10.7717/peerj.20655/supp-5 Supplemental Information 5 Supplemental figures and algorithm

## Additional Information and Declarations

### Data Availability

The following information was supplied regarding data availability: The raw data and code are available in the Supplemental Files.
