# Supplementary material for: Spatial validation of acoustic individual identification models without ground truths: a case study with the cao-vit gibbon population
Source: PeerJ. 2026 Mar 2;14:e20655. doi: 10.7717/peerj.20655 (PMC12962132; doi:10.7717/peerj.20655)
Supplement: Supplemental Information 5 [file peerj-14-20655-s005.pdf]

Song build-up phase detection for filtering

A challenge that comes with identifying individual males from ‘cao’ vocalisations is that some of their vocalisation are highly variable. Such variability hinders the quality of the clustering procedure (aiming at grouping ‘caos’ by individuals), since it reaches a point of overlap between frequency contour shapes of distinct individuals. This is especially the case for the beginning of songs, called song build-ups (Wearn et al., 2024) (supplementary Fig. S3). On that account, ‘caos’ that belonged to song build-ups were discarded for the clustering analysis. Following Wearn et al. (2024), we considered all ‘caos’ occurring in the first 8 min of a song to be part of the song build-up phase (caos detected from the same station and less than 1 min apart were considered from the same song).

It is worth noting that sometimes, several males are heard singing simultaneously or with intertwined vocalisations. With such simple sequence extraction method, overlapping songs will be considered from the same sequence. Hence, we are unable to assume that vocalisations of a given sequence are from the same male, which would have strongly helped in both developing and validating the AIID system. Moreover, if a song overlaps with another one that started long before, some vocalisations of the build-up phase will not be considered as such. This will lead to over-pessimistic results in the validation of the AIID model.

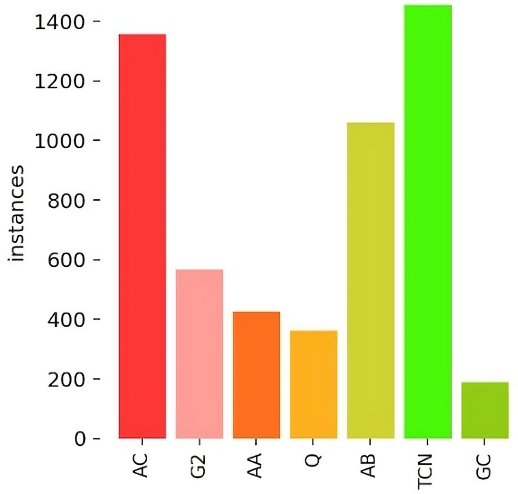

Figure S1. Distribution of labels for the classifier’s training (number of vocalisations per male gibbon)

Algorithm 1 Procedure to compute the consistency and specificity metrics from a set of localised detections with attributed identities.

$consistents \leftarrow 0$
$specifics \leftarrow 0$
for $call$ `in` $detections$ **do**
  $closeCalls \leftarrow detections[distance(detections, call) < threshold]$
  if $ID(call) == mode(ID(closeCalls))$ **then**
    $consistents \leftarrow consistents + 1$
  end if
  $sameInCloseCalls \leftarrow count(ID(call) == ID(closeCalls))$
  $distantCalls \leftarrow detections[distance(detections, call) > threshold]$
  $sameInDistantCalls \leftarrow count(ID(call) == ID(distantCalls))$
  if $sameInCloseCalls > sameInDistantCalls$ **then**
    $specifics \leftarrow specifics + 1$
  end if
end for
$specificity \leftarrow \frac{specific}{size(detections)}$
$consistency \leftarrow \frac{consistent}{size(detections)}$

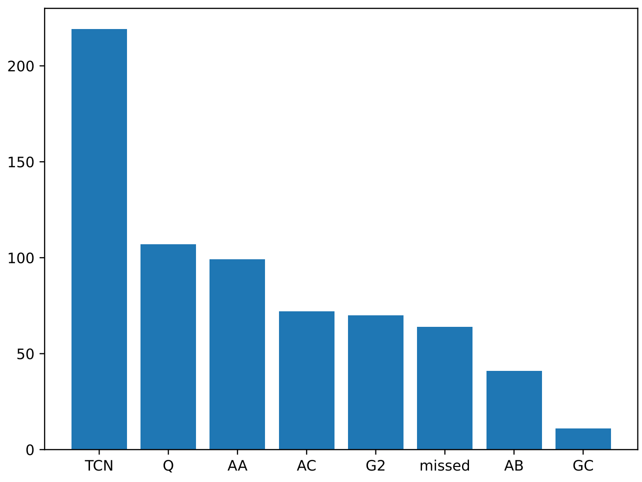

Figure S2. Distribution of male attributions for the multilaterated vocalisations. We applied the male ID YOLO model to multilaterated calls to attribute identities. The 'missed' entry corresponds to multilaterated calls over which the model did not detect anything.

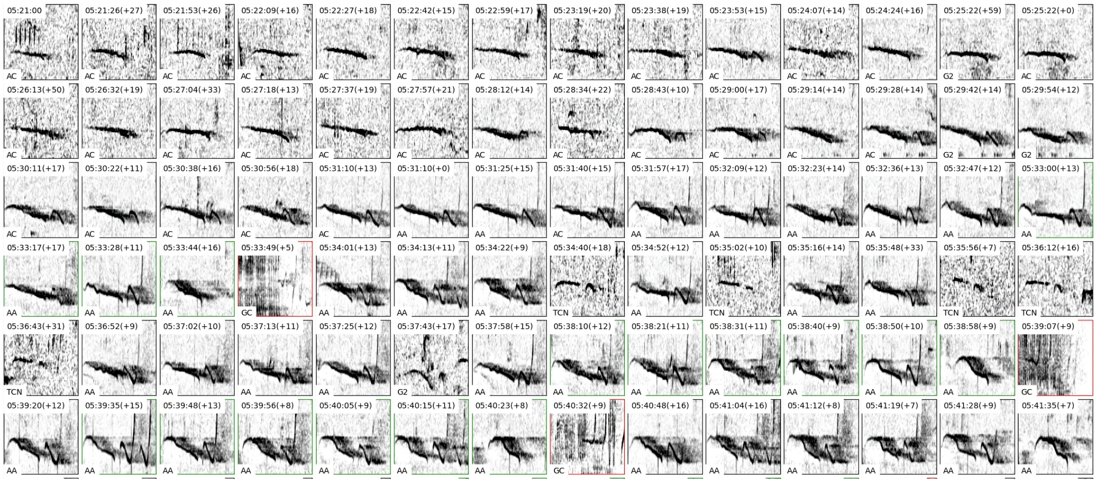

Figure S3. **Demonstration of the variability present in the build-up phase of a song, and its effect on the automated classification.** To ease the reading of this song, we display a grid of spectrograms for detected ‘caos’ (from left to right and top to bottom), removing the large silences between them. Here, the build-up phase can be observed in the first two rows. ‘Caos’ are shown with their date of detection, the time difference with the previous cao, and the detected class.
